# Model-based regulation of glucose in critical care

**DOI:** 10.1186/cc10785

**Published:** 2012-03-20

**Authors:** SP Gawel, G Clermont, RS Parker

**Affiliations:** 1 University of Pittsburgh, PA, USA; 2 University of Pittsburgh Medical Center, Pittsburgh, PA, USA

## Introduction

Glucose control in critical care has been shown to improve patient outcome, yet tight glucose control has led to increased hypoglycemia in the clinic. We employed a systems engineering approach to assist clinicians in maintaining blood glucose within a desired target range while avoiding hypoglycemia in the critically ill. The long-term vision is a decision support system that provides recommended insulin and glucose administrations leading to patient-specific achievement of tight glucose control without hypoglycemia.

## Methods

To achieve these goals, we employ a model predictive control (MPC) algorithmic platform using two control inputs: insulin for glucose control and glucose for hypoglycemia. The MPC controller is designed based on a nonlinear dynamic model of glucose-insulin-fatty acid interactions [1]. A moving horizon estimation (MHE) technique is used to alter the tissue sensitivity to insulin based on deviations between measurements and model predictions of glucose concentration as a mechanism for tailoring the controller model to individual patient dynamics.

## Results

**Figure 1:**
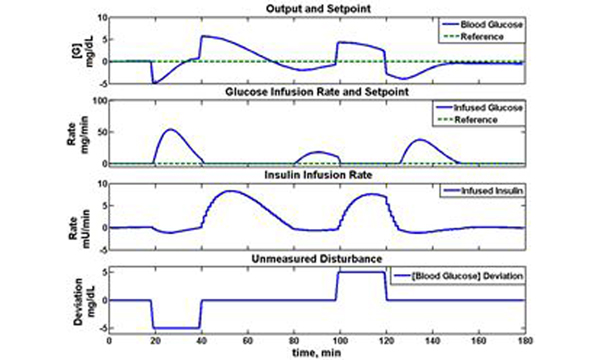


The response of the MPC controller to measured deviations in glucose is shown in Figure 1. For glucose measurements below target, glucose is administered, while insulin administration is used to lower blood glucose from an elevated state to a desired target. The model parameter pG2, representing patient insulin sensitivity (insulin action on glucose uptake), was used by the MHE algorithm to tailor the model response to simulated patient dynamics. In response to pG2 changes in the simulated patient, MHE provided a 93% improvement in glucose reference tracking performance.

## Conclusion

The algorithm achieves tight glucose control in response to multiple measured and unmeasured disturbances. Furthermore, the MHE scheme updates patient parameters in real time in response to changing patient dynamics. The adaptive MPC algorithm is currently being validated using a retrospective cohort of critically ill patients at the University of Pittsburgh Medical Center.
